# Relaxin reverses maladaptive remodeling of the aged heart through Wnt-signaling

**DOI:** 10.1038/s41598-019-53867-y

**Published:** 2019-12-06

**Authors:** Brian Martin, Beth Gabris, Amr F. Barakat, Brian L. Henry, Marianna Giannini, Rajiv P. Reddy, Xuewen Wang, Guillermo Romero, Guy Salama

**Affiliations:** 1 Department of Bioengineering, University of Pittsburgh, Pittsburgh, PA 15261 USA; 2 Department of Medicine, Heart and Vascular Institute, University of Pittsburgh, Pittsburgh, PA 15261 USA; 3 Department of Pharmacology & Chemical Biology, University of Pittsburgh, Pittsburgh, PA 15261 USA; 4 Scuola Superiore Sant’ Anna, Institute of Life Sciences, Pisa, Italy; 5 Present Address: Tianjin Key Laboratory of Ionic-Molecular Function of Cardiovascular disease, Department of Cardiology, Tianjin Institute of Cardiology, Second Hospital of Tianjin Medical University, Tianjin, 300211 China

**Keywords:** Ion channel signalling, Ion channel signalling, Ageing, Ageing

## Abstract

Healthy aging results in cardiac structural and electrical remodeling that increases susceptibility to cardiovascular diseases. Relaxin, an insulin-like hormone, suppresses atrial fibrillation, inflammation and fibrosis in aged rats but the mechanisms-of-action are unknown. Here we show that relaxin treatment of aged rats reverses pathological electrical remodeling (increasing Nav1.5 expression and localization of Connexin43 to intercalated disks) by activating canonical Wnt signaling. In isolated adult ventricular myocytes, relaxin upregulated Nav1.5 (EC_50_ = 1.3 nM) by a mechanism inhibited by the addition of Dickkopf-1. Furthermore, relaxin increased the levels of connexin43, Wnt1, and cytosolic and nuclear β-catenin. Treatment with Wnt1 or CHIR-99021 (a GSK3β inhibitor) mimicked the relaxin effects. In isolated fibroblasts, relaxin blocked TGFβ-induced collagen elevation in a Wnt dependent manner. These findings demonstrate a close interplay between relaxin and Wnt-signaling resulting in myocardial remodeling and reveals a fundamental mechanism of great therapeutic potential.

## Introduction

Aging is associated with structural and functional changes of the cardiovascular system and is a major risk factor for a number of pathologies, including atrial fibrillation (AF) and heart failure (HF)^1,2^. The prevalence of age-associated cardiovascular disease (CD) is expected to increase steadily, with estimates reaching 70 million people in the United States by 2030^1^. Structural and electrical remodeling during aging includes excess fibrosis, cellular hypertrophy and vascular stiffening leading to hypertension, reduced myocyte contractility, diastolic and/or systolic dysfunction and HF^1,3,4^. Age-dependent electrical remodeling involves ion channel and gap junction dysregulation which result in increased arrhythmia susceptibility^4–7^. We previously showed that the hormone relaxin (RLX) suppressed AF in aged (24-months old) rats by increasing conduction velocity (CV) of atrial action potentials^5^. These effects were linked to increased expression of the voltage-gated sodium channel Nav1.5, increased current (I_Na_), and a marked decrease in fibrosis^5^. However, the mechanisms whereby RLX acts to increase Nav1.5 and reduce fibrosis are largely unknown, though studies indicate that RLX suppresses TGFβ and matrix metalloproteases in fibrosis regulation^5,6,8^. We reported that RLX-treatment of young (9-months) and aged (24-months) rat (F-344 strain) ventricles resulted in a reversal of inflammatory and immune responses and an overall rejuvenation of cardiac properties^9^.

Canonical Wnt signaling is a master controller in cardiac embryogenesis and development, but it is thought to be quiescent after birth^10,11^. In the absence of Wnt signaling, β-catenin is phosphorylated, marked for ubiquitination and removed by the proteasome. During canonical Wnt-signaling, Wnt binding to Frizzled and the co-receptors, LRP5/6, results in the dissociation of the β-catenin destruction complex causing the accumulation of β-catenin, which enters the nucleus to regulate the activity of the TCF/LEF family of transcription factors. Exogenous Wnt3a is routinely added to stimulate the differentiation of human induced pluripotent or embryonic stem cells into cardiac myocytes and conversely, blockade of Wnt-signaling with Dickkopf-1 (Dkk1) inhibits cardiac differentiation^12^. Interestingly, late antagonism of endogenous Wnts enhances cardiogenesis, indicating a biphasic role in human cardiac differentiation^12,13^. Besides the regulation of the contractile apparatus, Wnt-signaling regulates the expression of the voltage-gated Na^+^ channel Nav1.5 in neonate rat ventricular cardiomyocytes (NRVM)^11^ and HL-1 cells^14^. Puzzlingly, Wnt3a suppressed Nav1.5 expression in NRVM^11^, whereas activation of canonical Wnt signaling by other means increased Nav1.5 and Connexin-43 (Cx43), and reduced ventricular ectopic beats in NRVM, 3-week and 6-month old mouse models of arrhythmogenic cardiomyopathy^15–17^.

Here, we tested the hypothesis that the effects of RLX on Nav1.5 and fibrosis are mediated by canonical Wnt signaling. While RLX and Wnt signaling have been investigated in models of cancer^18–20^, there is no work on the interactions of the RLX and Wnt pathways in adult heart and ‘healthy’ aging as a precursor of cardiac diseases. We focused on hearts from 9-month (adult) and 24-month-old (aged) Fischer 344/Brown Norway F1 (F-344) male rats obtained from the National Institute of Aging. F-344 rats have been extensively studied and characterized as a model of aging because of their linear aging, and because, like humans, they exhibit an age-dependent excess of cardiac fibrosis and susceptibility to atrial fibrillation^5,21^. Rats were treated with RLX (400 μg/kg/day) or vehicle (sodium acetate) via subcutaneous osmotic mini-pumps for 14 days, resulting in an increase in the circulating plasma concentration of RLX from 0.017 ± 0.027 ng/mL to 23.76 ± 10.60 ng/mL, as determined by an ELISA assay.

## Relaxin Improves Ventricular Function

**Figure 1:**
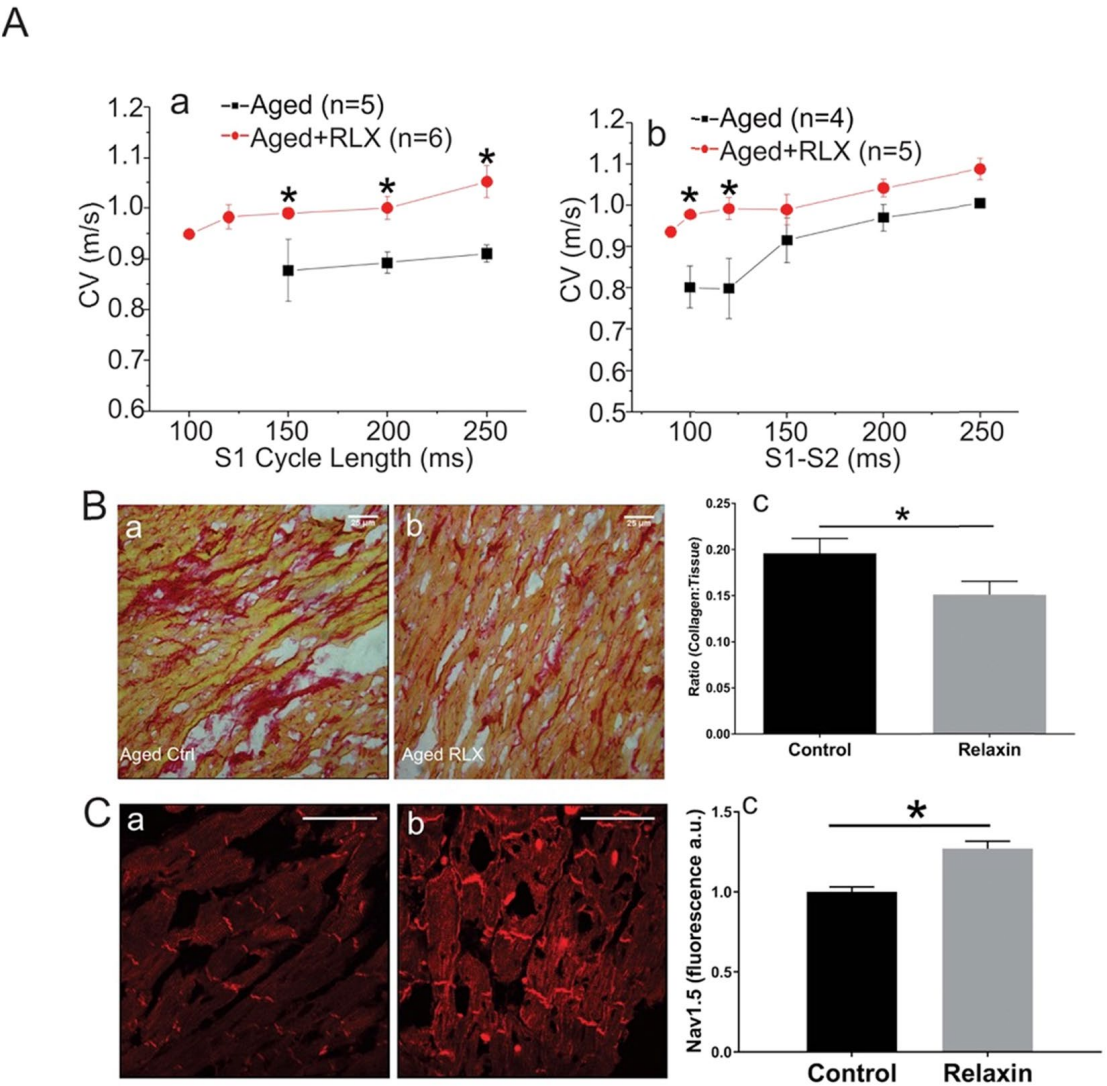
Relaxin increases CV and reduces collagen in aged LV. (**A**) (a) RLX treatment resulted in a significant increase in CV during steady pacing (S1:S1). (b) CV restitution kinetics measured by programmed stimulation with premature pulses (S2 beats) was significantly increased by RLX, particularly during fast pacing times. *Indicates *P* < 0.05. 20x magnification (**B**) LV tissue sections were stained with Picro-sirius red and imaged on an Olympus Provis Light Microscope. The collagen to tissue ratio was significantly reduced in RLX (Bb, n = 6) treated animals compared to control (Ba, n = 5) by 23%. (**C**) LV tissue stained for Nav1.5 showed that RLX (Cb, n = 6) significantly increased Nav1.5 expression compared to control hearts (Ca, n = 5). 600x magnification, scale bars = 25 µm and apply to all panels.

Hearts were perfused in a Langendorff apparatus for optical mapping of atrial and ventricular action potentials to calculate conduction velocity (CV)^5,6^. Aged ventricles treated with RLX showed a marked increase of CV, an effect that was particularly pronounced at fast pacing rates compared to aged control ventricles (Fig. 1A*a*). CV restitution kinetics measured by programmed stimulation revealed that premature stimuli elicited depolarizing waves in control aged rats with considerably slower CV than in RLX treated aged-rats (Fig. 1Ab). It is interesting to note that when paced at cycle lengths below 150 ms, control aged ventricles failed to capture and propagate or maintain a steady state CV whereas RLX-treated ventricles could be paced at those faster rates without significant decrement of CV (Fig. 1Aa). In ventricles of RLX treated rats, the slope of the restitution kinetics curve becomes less steep than for untreated animals which suggests a decrease in arrhythmia vulnerability (Fig. 1Ab).

A hallmark of advancing age is an escalation of excess fibrosis. Increased collagen deposits are associated with cardiac dysfunction due to disruption of cell-cell coupling and a depolarization of the resting membrane potential^22^. Collagen content was measured by staining left ventricular (LV) sections with Picro-Sirius Red and by calculating the collagen to tissue ratio in RLX treated and control animals. RLX treatment significantly reduced the collagen to tissue ratio (Fig. 1Ba–c). Most important, RLX reversed age-associated fibrosis to levels like those measured in 11-month-old rats^23^.

In addition, reductions in Nav1.5 expression contribute to arrhythmia and other heart pathologies by slowing CV^22^. RLX significantly increased Nav1.5 expression in LV sections compared to control (Fig. 1Ca–c).

**Figure 2:**
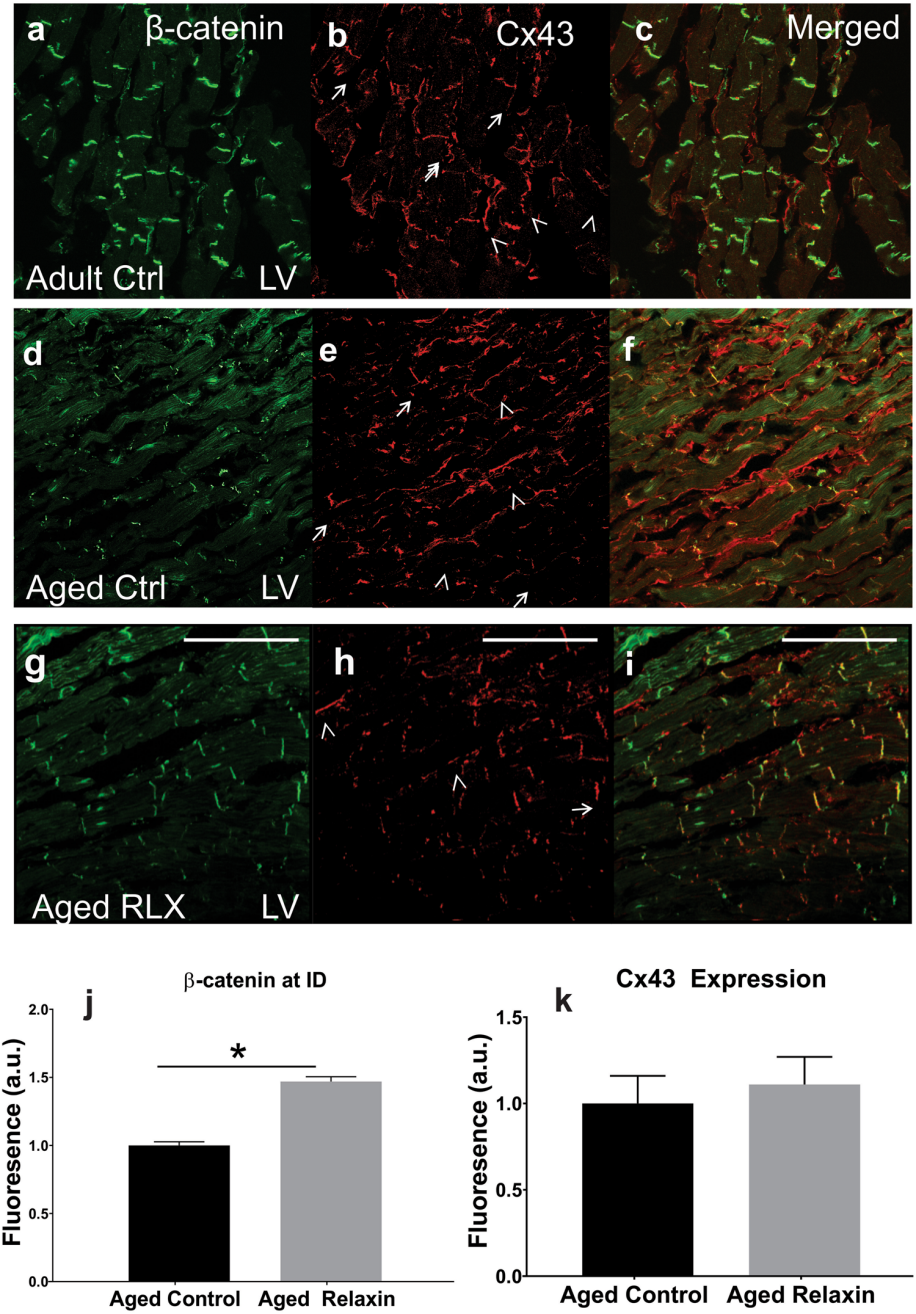
Age and relaxin’s effect on intercalated disk proteins in LV. In adult (9-months, n = 4) F-344 rats (**A**a–c), β-catenin and Cx43 localized to the intercalated disks (arrows), with minimal movement of Cx43 to the lateral membranes (arrow heads). Untreated aged (24-months, n = 4) rats (**A**d–f) exhibited a marked reduction in β-catenin and translocation of Cx43 to the lateral membranes. RLX treatment (n = 4) of aged animals (**A**g–i) showed that RLX can reverse the effects of aging on β-catenin expression and results in trafficking of Cx43 to the intercalated disk, matching closely that seen in 9-month-old rats. (**A**j–k). Quantification of β-catenin/Cx43 expression. 600x magnification, scale bars = 25 µm and apply to all panels.

**Figure 3:**
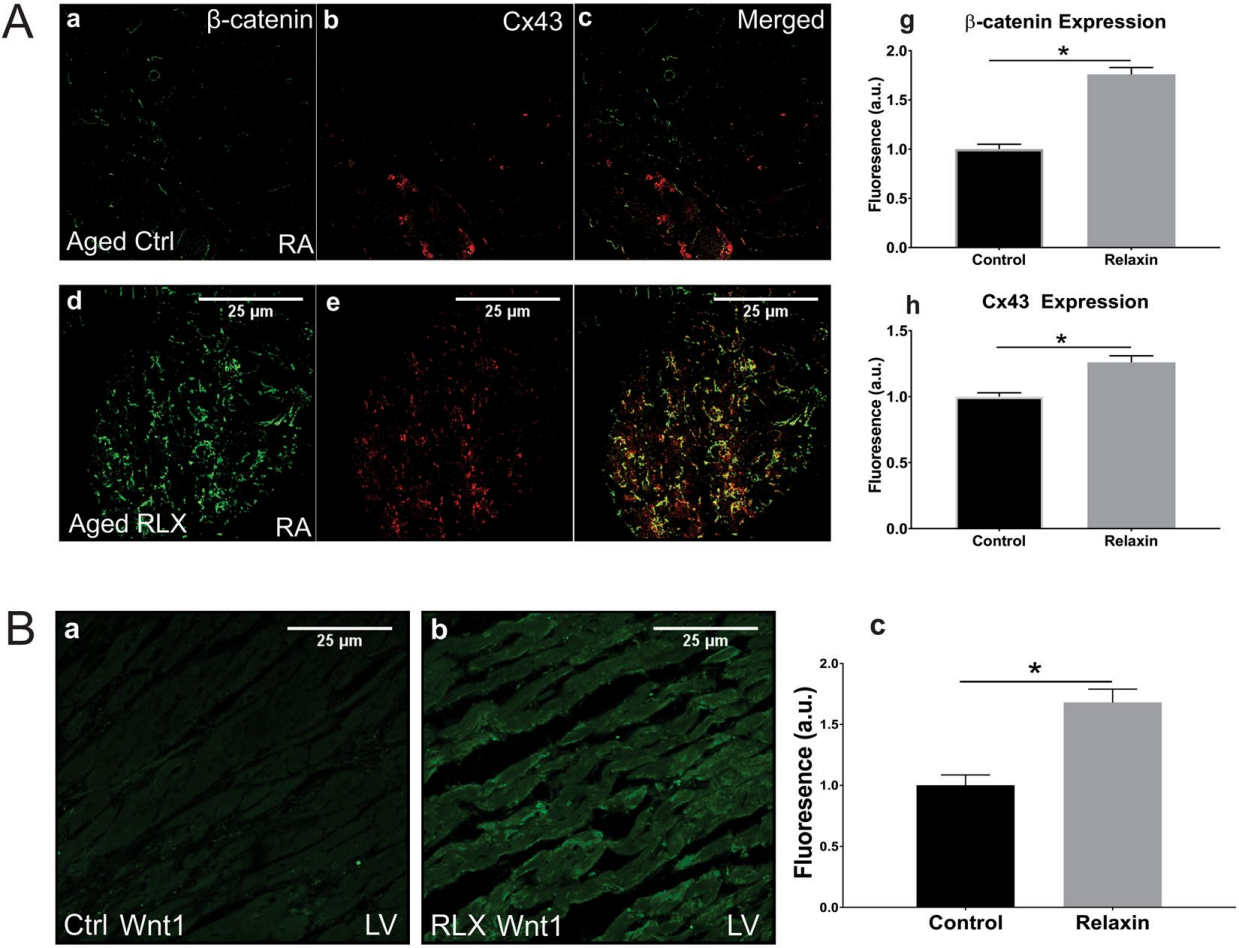
Age and relaxin’s effect on the levels of Cx-43 and β-catenin in aged atria. (**A**a–c) Aged right atrial tissue (**A**a–c) expressed significantly less β-catenin and Cx43 than RLX-treated animals (**A**d–f). (**A**g–h) Quantification of atrial β-catenin and Cx43 expression (n = 5/group). 60x magnification. (**B**) RLX (**B**b) increased Wnt1 expression in LV compared to control (**B**a). (**B**c) Quantification of Wnt1 expression in LV sections. 600x magnification, scale bars = 25 µm and apply to all panels.

Further, β-catenin was reduced in LV sections of aged (Fig. 2d) compared to adult (Fig. 2a) rat hearts and was diffusely distributed in the cytosol with relatively little localization at intercalated discs (IDs) while Cx43 was localized to the lateral membranes in aged vs adult LV (Fig. 2e vs. 2b). RLX shifted the localization of Cx43 from the lateral membranes to IDs, increased the levels of β-catenin, and β-catenin became co-localized with Cx43 at IDs (Fig. 2g-i). In general, LV of aged-rats with RLX-treatment resembled the LV of adult rats (Fig. 2a–c). This strongly suggested that RLX treatment reversed some of the aging-induced changes observed in 24-month-old rats. Similar findings were seen in aged atria (Fig. 3A). Control experiments showed that the same Relaxin treatment on young (9 months old) rats had no significant effects, see Supplemental Fig. S1.

## Relaxin Acts Via Wnt-Signaling in Aged Rats

**Figure 4:**
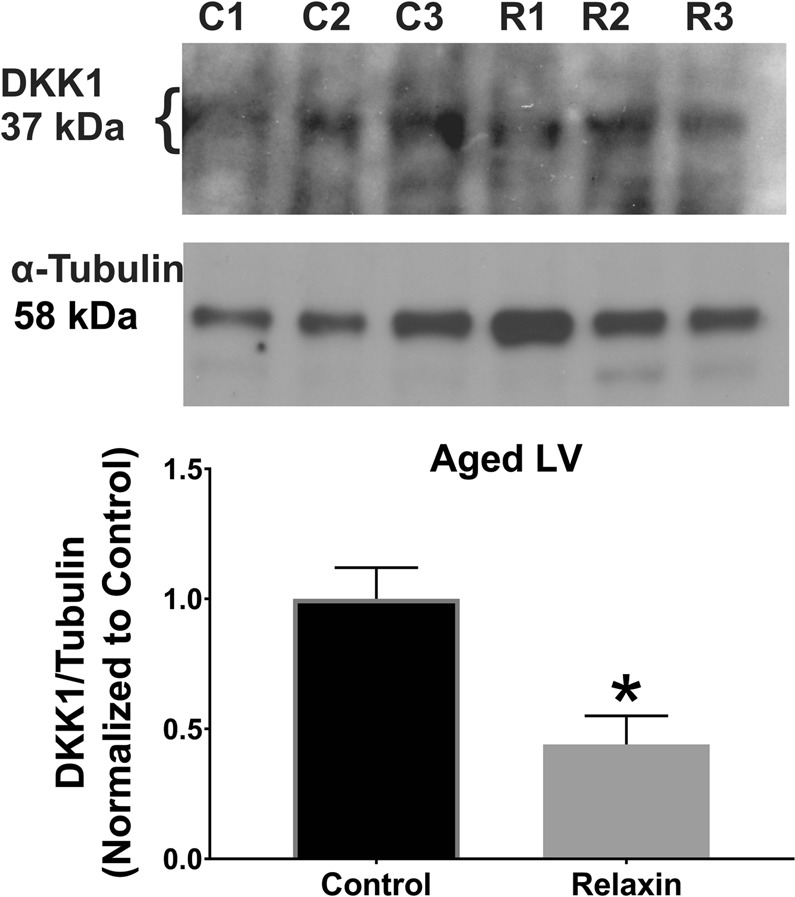
Relaxin inhibits DKK1 expression in Aged LV. Relaxin significantly reduced DKK1 expression compared to control hearts. C = control. R = Relaxin. n = 3/group. Gels were probed for DKK1 expression (top), stripped and re-probed for, and normalized to, α-tubulin expression (bottom). Line delineates separate probe experiments, measured on the same membrane. For full blots, see Supplementary Fig. S2.

Based on previous work of Wnt signaling effects on Nav1.5 and ID proteins, we tested the role of Wnt-signaling in RLX-mediated upregulation of Nav1.5 in adult and aged animals. RNA from aged atria ± RLX-treatment followed by RT-PCR showed the expected reduction of fibrosis mRNA biomarkers (Metalloproteinases (MMPs: MMP-2 and 9, α-smooth muscle actin) but also showed a marked increase in Wnt1 expression (Fig. 3, Table 1). Likewise, RLX-treatment increased Wnt1 expression in LV sections (Fig. 3B). Next, tissue from aged LV ± RLX was tested for Dickkopf-1 (Dkk1) expression, an intrinsic inhibitor of Wnt signaling. Dkk1 has been shown to bind LRP6 and blocks canonical signaling of all Wnt ligands. RLX-treated LV showed a significant reduction of Dkk1 compared to control LV (Fig. 4). These data suggest that RLX acts to activate Wnt signaling by two complementary mechanisms: increasing Wnt1 expression and reducing Dkk1 and Wnt inhibition.

**Table 1:** Fibrosis and Wnt-related gene expression for RLX treated atria.

Protein	Gene	Expression	n/group	p
MMP-2	MMP2	−43 ± 13%	8	0.004
MMP-9	MMP9	−41 ± 17%	18	0.02
αSMA	ACTA2	−27 ± 18%	18	0.04
Wnt-1	WNT1	+80 ± 16%	18	0.01

*Relative to aged control.

Relaxin altered expression of mRNA in aged atria that relate to fibrotic and Wnt related genes. Data were obtained by quantitative RT-PCR as described under Methods. Data are reported as percent change of mRNA levels obtained from RLX-treated aged atria relative to vehicle-treated aged controls ± standard deviation (n = number of rats/hearts per group).

### RLX-Wnt Interaction in Isolated Cardiomyocytes

**Figure 5:**
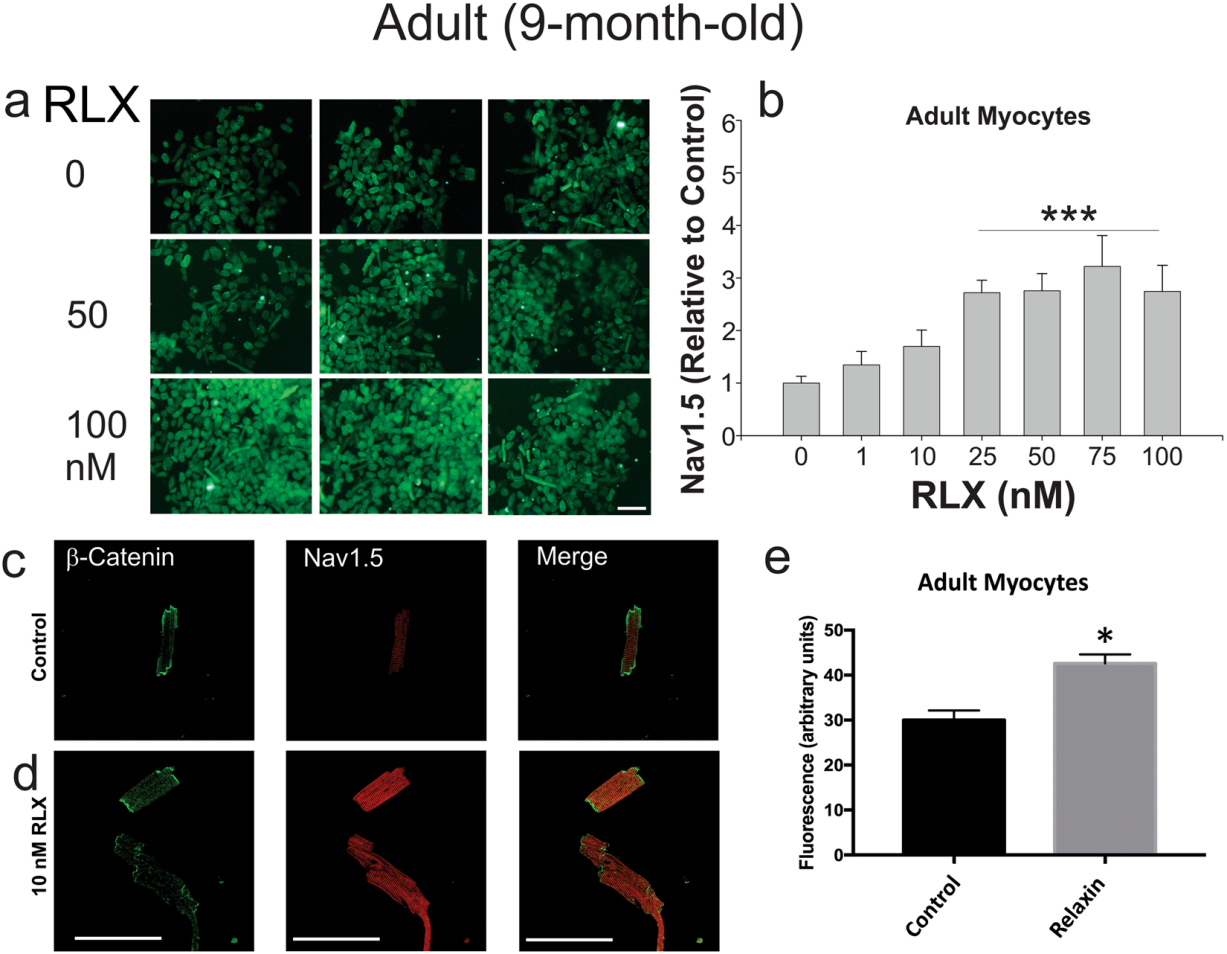
Relaxin up-regulates Nav1.5 in LV cardiomyocytes. (**A**) Rat ventricular myocytes treated with increasing concentrations of RLX were fixed and labeled with Nav1.5 Ab. The data show that Nav1.5 fluorescence visibly increases with RLX treatment. (**B**) Dose-response of the ratio of Nav1.5 to phalloidin fluorescence (used for normalization purposes). EC_50_~1.3 nM, (*n* = 6 wells/data point, *P* < 0.01). (**C**,**D**) RLX significantly increased Nav1.5 expression in isolated myocytes compared to control (*n* = 10–12 cells/group; 3 replicate experiments). 600x magnification, scale bars (25 µm) apply to all panels.

The effects of RLX were studied in isolated adult left ventricular myocytes by culturing the cells in 96 well plates with various doses (0–100 nM) of RLX for 2-days (Fig. 5a,b). After treatment, the myocytes were fixed, labeled with Nav1.5 antibody and fluorescein-conjugated secondary antibody and fluorescent phalloidin (to label actin used as a counterstain for normalization purposes). Fluorescence intensities showed that RLX up-regulated Nav1.5 with an EC_50_ of 1.3 nM (Fig. 5c–e), which is consistent with the reported affinity of RLX for its cognate receptor, RXFP1^24^.

**Figure 6:**
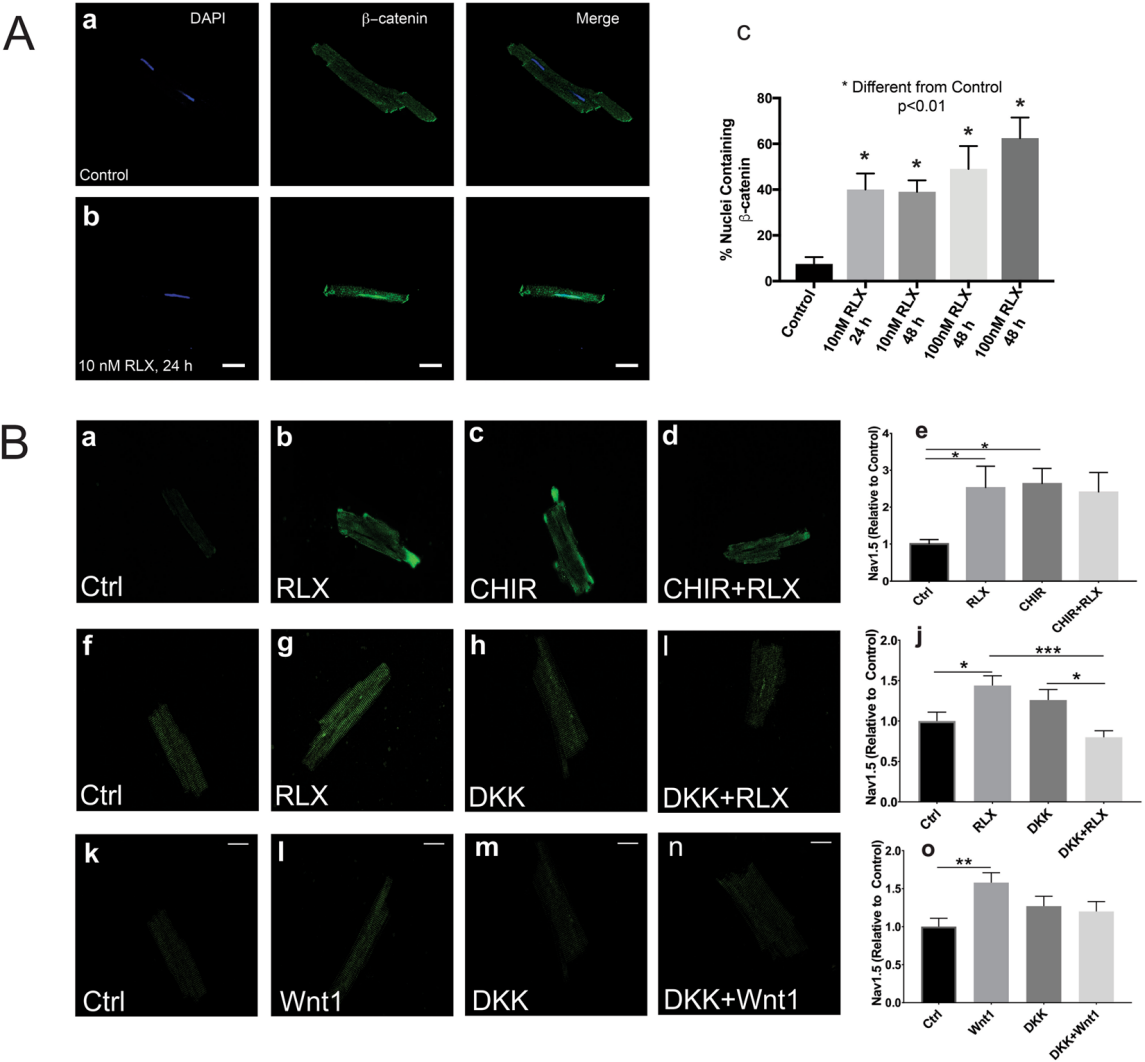
Relaxin signals through Wnt signaling to increase Nav1.5. (**A**) Less than 10% of untreated myocytes exhibit nuclear β-catenin (**A**a,c); however, cells treated with RLX for 24 or 48 hours showed a significant increase in cells positive for nuclear β-catenin (**A**b,c). 60x magnification. Data obtained from 3–4 separate preparations, 62–148 cells counted per sample. (**B**) Cells treated with RLX or CHIR, an inhibitor of GSK3β and Wnt pathway activator, significantly increased Nav1.5 by more than 2-fold (**B**a–e, n ≥ 8 cells/group). Inhibition of canonical Wnt signaling by DKK1 blocked the effects of RLX 9 n ≥ 24 cells/group) and Wnt1 (n ≥ 17 cells/group) on Nav1.5 expression (**B**f–o). 600x magnification, scale bars = 25 µm and apply to all panels. *Indicates p < 0.05 compared to controls. *Indicates p < 0.05. **Indicates p < 0.01. ***Indicates p < 0.001.

As shown in Fig. 2, RLX increased β-catenin and Cx43 trafficking to the ID. To determine whether RLX also acted via a Wnt-related mechanism in isolated myocytes, we incubated freshly isolated ventricular myocytes with RLX (25 nM; 24–48 hours) or vehicle (Na^+^ acetate) and then double stained with DAPI (nuclear stain) and a β-catenin antibody to measure the number of myocytes that expressed nuclear β-catenin. Control myocytes had a considerably smaller fraction of β-catenin-containing nuclei (Fig. 6Aa,c) compared to RLX treated myocytes (Fig. 6Ab,c), suggesting that RLX is acting in part by activating canonical Wnt signaling in isolated cardiomyocytes.

To determine if Wnt signaling is directly involved in Nav1.5 regulation, myocytes were treated with either CHIR-99021 (a GSK3β inhibitor, 3 µg/mL), RLX (25 nM) ± Dkk1 (0.1 µg/mL) or Wnt1 (0.1 µg/mL) ± Dkk1 for 24 hours. Activation of Wnt signaling by the GSK3β inhibitor, CHIR-99021 resulted in a significant increase in Nav1.5 expression to the same extent as RLX alone and compared to control myocytes. The simultaneous treatment of myocytes with CHIR-99021 and RLX did not result in a further increase of Nav1.5 expression (Fig. 6Ba–e*)*. Inhibition of Wnt signaling by Dkk1 significantly blocked RLX (Fig. 6f–j) and Wnt1’s (Fig. 6Bk–o) effects on Nav1.5 expression. These results strongly suggest that, contrary to the effects reported in NRVM, canonical Wnt signaling in adult cardiomyocytes increases Nav1.5 expression.

**Figure 7:**
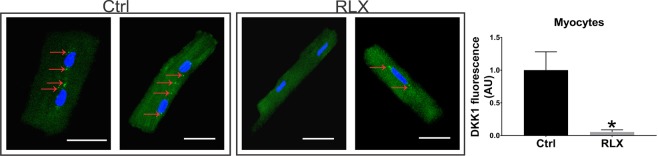
Relaxin inhibits DKK1 expression in isolated cardiomyocytes. Relaxin treatment for 2-days significantly reduced expression of DKK1 in isolated cardiomyocytes compared to control cells (n ≥ 14 cells/group). *Indicates p < 0.05. White lines = 25 µm.

The effect of RLX on Dkk1 seen in rats treated with the hormone was also studied in isolated cardiomyocytes by treating the cultured myocytes with Na-acetate (control) or RLX for 2-days followed by labeling with an anti-Dkk1 antibody. In control myocytes, Dkk1 protein appeared as broadly distributed punctate spots inside the cells (Fig. 7 arrows)^25^. In contrast, RLX treatment significantly reduced the expression of Dkk1 in cardiomyocytes (Fig. 7). Thus, at least in part, RLX activates Wnt signaling in both cardiomyocytes and LV tissue through a downregulation of constitutively expressed DKK1.

## Interplay Between RLX and Wnt in Fibroblasts

**Figure 8:**
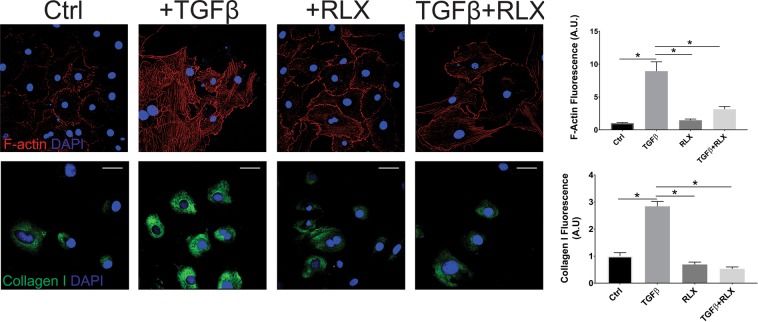
Relaxin blocks fibroblast to myofibroblast transition. Fibroblasts treated with TGFβ showed a marked increase in F-actin (top, n ≥ 53 cells/group) and collagen I (bottom, n ≥ 41 cells/group) expression that was dramatically inhibited by RLX. *Indicates p < 0.05. 400x magnification, scale bars = 25 µm and apply to all panels.

**Figure 9:**
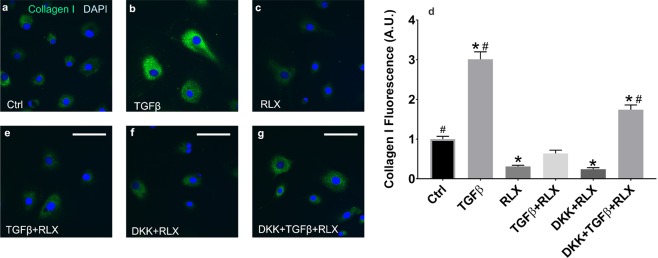
Relaxin-Wnt interaction modulates collagen secretion by Fibroblasts. Fibroblasts treated with TGFβ showed a 3-fold increase in collagen expression that was dramatically inhibited by RLX to 31% of control expression.(**a**–**d**) DKK1 blocked RLX’s inhibitory effects of TGFβ mediated collagen expression (**d–g**); n ≥ 43 cells/group. *Indicates p < 0.05 compared to control. ^#^Indicates p < 0.05 compared to RLX. 400x magnification, scale bars = 25 µm and apply to all panels.

**Figure 10:**
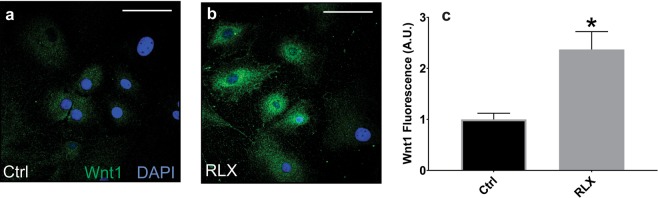
Relaxin alters Wnt1 protein expression in cardiac fibroblasts. Relaxin significantly increased expression of Wnt1 in cardiac fibroblasts. *Indicates p < 0.05. n ≥ 46 cells/group. 400x magnification, scale bars = 25 µm and apply to all panels.

Fibrosis is a major contributor to impaired cardiovascular function and diastolic heart failure and has been shown to increase with age^23^. We have shown that RLX significantly reduces fibrosis in the atria^5^ and ventricles of aged rats (Fig. 1). TGFβ significantly increased the differentiation of fibroblasts into myofibroblasts as determined by the increased levels of collagen I and F-actin, and RLX blocked these effects (Fig. 8). The suppression of the differentiation of fibroblasts to myofibroblasts by RLX has been reported in neonate or immortalized cell lines^8,26^ but these cells do not necessarily respond to RLX in the same manner as adult primary cardiac fibroblasts and cannot be used as evidence of the anti-fibrotic actions of RLX in animals. To determine the role of Wnt signaling on the effects of RLX in cardiac fibroblasts, adult F-344 cardiac fibroblasts were isolated, cultured with RLX and/or TGFβ and were treated with recombinant Dkk1 for 72 hours, after which, collagen levels were measured by immunofluorescence. TGFβ significantly increased collagen compared to control (Fig. 9a,b) and RLX blocked TGFβ’s effects (Fig. 9b). The addition of Dkk1 resulted in a significant reversal of the effects of RLX on collagen expression (Fig. 9g). Dkk1 had no effects on RLX alone (Fig. 9f). These data are summarized in the bar graph shown in Fig. 9d. These findings are consistent with RLX’s action in ventricular myocardium. Furthermore, RLX increased the expression of Wnt1 in cardiac fibroblasts (Fig. 10). These data strongly support our hypothesis: RLX increases activation of canonical Wnt signaling, and the effects of RLX on gene expression and TGFβ signaling are significantly inhibited by the canonical Wnt signaling inhibitor Dkk1.

## Discussion

Based on intriguing clinical and pre-clinical data, RLX engendered significant enthusiasm as a potential therapy for cardiopulmonary diseases. In the acute heart failure trial RELAX-AHF, RLX treatment (30 µg/kg/day for 2-days, i.v.) improved patient survival by a remarkable 37% in 6 months^27^. These exciting results led the FDA to declare RLX as a “break-through” therapy made all the more significant because the trial included patients with systolic and diastolic HF. Unfortunately, the reduced mortality benefits were not duplicated in a subsequent larger clinical trial (www.novartis.com). Detailed analysis of the larger trial has not been reported and the failure of RLX to significantly reduce mortality is not fully understood. A possible explanation is that the control group of patients receiving standard of care for HF fared considerably better than in earlier trials but another problem has been the design methodology of a 2-days treatment which has justifiably received substantial criticism^28^. Our previous studies on the effects of RLX in experimental animals provide compelling evidence of significant beneficial effects of the hormone in cardiac physiology. We reported that RLX suppressed AF in aged (24-months old) rats by increasing conduction velocity (CV) of atrial action potentials^5^. These effects were linked to increased expression of the voltage-gated sodium channel (Nav1.5) and current (I_Na_), and a marked decrease in fibrosis^5^, both effects confirmed here in ventricles. At the cellular level, Relaxin was shown to upregulate I_Na_ in 24-hours but the reversal of fibrosis required more than a week due to the slow turn-over of collagen in the extracellular matrix^6^. Besides electrical and ECM remodeling, we reported that Relaxin acted as a potent anti-immune and anti-inflammatory agent in the ventricles of aged animals^2^. Here we examine the mechanisms responsible for RLX’s functions in the heart. Our results show that RLX’s effects are largely mediated by the modulation of canonical Wnt signaling which can act as a master controller of gene expression in heart and other organs.

This conclusion is based on several major lines of evidence: a) GSK3β inhibitors and exogenous Wnt1 protein mimic the effects of RLX in cardiomyocytes; b) recombinant Dkk1, a specific natural inhibitor of canonical Wnt signaling, blocks the effects of RLX on Nav1.5 and collagen I expression; c) Dkk1 expression is reduced by RLX in LV tissue and isolated cardiomyocytes; and d) RLX increases the expression of the Wnt1 ligand in cardiac fibroblasts. The integration of RLX and Wnt signaling was previously suggested by studies in prostate cancer^18^, but the mechanisms described here are very different. Whereas in prostate cancer RLX promotes tumor progression via its effects on the protocadherin PCDHY^18^, the effects we described are mediated, at least in part, by increased expression of Wnt1 and reduced expression of Dkk1.

Wnt signaling in the heart is complex, and different Wnt ligands have distinct effects. Previous studies of the actions of Wnt-signaling focused on isolated neonate rat ventricular myocytes (NRVM) and non-cardiac fibroblasts. In these earlier studies, treatment with Wnt1 and inhibition of GSK3β increased Cx43 expression and trafficking to the ID^15,16^, results that are consistent with those reported here. The role of Wnt signaling in the regulation of Nav1.5 is more controversial. Liang *et al*. reported that incubation of NRVM with Wnt3a or CHIR-99021 down-regulated Nav1.5^11^, results that were confirmed using the immortalized cardiomyocyte cell line HL-1^14^. In contrast, findings by Asimaki *et al*.^17^, Chelko *et al*.^16^ and data presented here, show that canonical Wnt signaling and GSK3β inhibition increase Nav1.5 expression in adult, aged, and diseased cardiomyocytes, suggesting that, like Wnt-signaling, RLX signaling is highly dependent on cell type and age.

Finally, many details of the mechanism by which RLX modulates canonical Wnt signaling remain to be explored. RXFP1 has been reported to signal via cAMP-dependent mechanisms downstream of the activation of heterotrimeric proteins of the G_s_, G_i_ and G_o_ families^29^. Our research, however, suggests that the effects of RLX on Nav1.5 expression are independent of cAMP signaling, since adenylate cyclase and PKA inhibitors did not alter the effects of RLX on the expression of Nav1.5 (data not shown). Furthermore, attempts to show RLX-dependent regulation of cAMP levels in cardiomyocytes using a sensitive colorimetric immunoassay were unsuccessful. Likewise, we failed to see any significant effects of RLX treatment on the levels of phosphorylation and/or nuclear translocation of CREB in isolated cardiomyocytes (not shown). Thus, we concluded that the substantial effects of RLX on Nav1.5 expression are not a consequence of the effects of RLX on cAMP signaling. RLX has also been reported to signal through a variety of pathways, including nitric oxide, PI3 Kinase, or via a relatively uncharacterized pathway that involves a direct interaction of RLX with the glucocorticoid receptor and the latter’s subsequent activation^30^. The involvement of GR-dependent pathways is unlikely given that GR signaling increases Dkk1 expression and blocks canonical Wnt signaling^31^. However, we cannot rule out at this point a mechanism mediated by the production of nitric oxide.

Note that there is no simple correlation between rats treated with Relaxin for 2-weeks with isolated cells incubated with Relaxin for 24–48 hours. Delivery of exogenous Relaxin systemically to a rat raises questions of dosing, duration, circulating levels of RLX and its actions at many targets that can alter hemodynamics, immune and inflammatory responses; not just the heart. Relaxin added to cells dissociated with collagenase raises questions about cellular damage by the isolation procedure and the brief survival of primary cell cultures, particularly aged cardiac myocytes which in our hands lasted less than 24 hours and could not be used to test a genomic mechanism. Nevertheless, the cellular experiments show that Relaxin acts at the cellular level to explain most of its actions on the heart and that the genomic changes on Nav1.5, connexin 43 and β-catenin occur within 24 hours. Hence, cell studies offer a reasonable option to elucidate the modes of action of Relaxin.

## Methods

### Animal study design

Twenty-four-month-old Fischer 344/Brown Norway F1 male rats from the National Institute of Health were separated into RLX (400 µg/ml/day, *n* = 6) and control (*Na*^+^ *acetate*, *n* = 5) groups and treated for 14 days via subcutaneous osmotic mini-pumps (Alzet® Cupertino, CA; model 2ML2). Relaxin was the generous gift of the RRCA Foundation (Foundation for Research on Relaxin in Cardiovascular and Other Diseases, Drs. Mario Bigazzi and Daniele Bani, Prosperius Institute, Florence, IT). Studies were performed in accordance with the Guide for the Care and Use of Laboratory Animals and were approved by the Institutional Animal Care and Use Committee at the University of Pittsburgh.

### Plasma relaxin concentration

Circulating levels of RLX in the aged rats were determined using a pre-packaged Quantikine ELISA kit (R&D Systems, DRL200) according to manufacturer instructions.

### Optical mapping

The optical mapping apparatus was described in detail in several reports^5,32^. Briefly, hearts were excised and perfused on a Langendorff apparatus with Tyrode’s solution containing (in mM): NaCl (130), KCl (4.5), KH_2_PO_4_ (0.6), Na_2_HP0_4_ (0.6), MgSO_4_ (1.2), HEPES (10), NaHCO_3_ (24), Glucose (50), gassed with 95% O_2_ and 5% CO_2_, pH 7.0 at 37 °C. Hearts were placed in a custom-designed chamber to abate motion artifacts and blebbistatin (5–7 µM) briefly added to the perfusate to minimize movement artifacts. Bolus injections of voltage (RH 237 50 µl of 1 mg/mL dimethyl sulfoxide (DMSO)) and Ca^2+^-indicator dye (Rhod-2/AM, 80 µl of 1 mg/mL DMSO) were made in the air-trap above the aortic cannula. Fluorescence from the epicardium was collected with a camera lens, split with a 570 nm dichroic mirror and focused on two CMOS cameras (Sci-Media UltimaOne) capturing at the fluorescence emission at 570–595 nm for cytosolic Ca^2+^ and 610–750 nm wavelengths for voltage. The hearts were paced at various cycle lengths (CL) and with programmed stimulation, starting with a baseline CL for 10 beats at S1-S1 = 250 ms followed by a single S2 pulse delivered at decreasing S1-S2 intervals. Pacing protocols were used to measure CV at various stable CLs (S1-S1) and CV restitution kinetics was measured through the CV of the premature impulse delivered at decreasing S1-S2, until the premature impulse failed to capture or triggered an arrhythmia.

### Immunohistochemistry

Tissue sections (7 μm) were treated with 0.1% Triton X-100 followed by block with 2% BSA. Primary antibody was added for 1 hour at room temperature. Guinea Pig and Rabbit anti-Nav1.5 (AGP-008, Alomone Labs, 1:200 in tissue and ab56240, Abcam, 1:200 in cells), rabbit anti-β-catenin (ab32572, Abcam, 1:250), mouse anti-Cx43 (sc-13558, Santa Cruz Biotechnology, Inc, 1:100), rabbit anti-collagen I (ab34710, Abcam, 1:200), mouse anti-Wnt1 (10C8, Thermo Fisher, 1:200) antibodies were used to measure protein expression. Secondary antibodies were applied for 1 hour at room temperature. Immunofluorescence imaging was performed using an Olympus Fluoview 1000 confocal microscope or an Olympus Provis light microscope. Isolated cardiomyocytes and fibroblasts were stained in a similar manner after fixation with 4% paraformaldehyde. In order to ensure quantitative reliability of the results, all samples being compared were immunostained simultaneously with the same antibody preparation, and identical confocal microscope settings were utilized for the recording of all data. NIH ImageJ was used for image analysis. Quantitation of fluorescence labeling was done after subtracting backgrounds using the built-in background subtraction function of ImageJ. To measure the fluorescence intensity of proteins associated to the intercalated disk, the cell-cell contact regions of adjacent cardiomyocytes were identified in the microscopic image, selected, and the intensities measured and recorded using ImageJ tools.

### Myocyte isolation

Excised hearts were placed on a Langendorff apparatus and perfused for 4 minutes with Perfusion Buffer containing (in mM): NaCl (130), KCl (14.7), KH2PO4 (0.6), Na2HP04 (0.6), MgSO4 (1.2), HEPES (10), NaHCO3 (4.6), Taurine (30), BDM (10), Glucose (5.5), pH 7.0. Heart was perfused for 11–14 minutes with digestion buffer containing: 50 mL Perfusion Buffer with 2 mg/mL Collagenase Type II. Heart was placed in small glass beaker with 3 mL digestion buffer and minced into small pieces with surgical scissors. Additional mincing was conducted by cutting plastic transfer pipettes at 45 degrees at largest diameter, slowing reducing pipette diameter size as tissue becomes solubilized. Stopping buffer (10 mL) was added containing: 45 mL Perfusion Buffer, 5 mL of 10% FBS and 12.5 µM CaCl_2_. Myocytes were transferred to a 50-mL tube through a cell strainer primed with stopping buffer and allowed to pellet (~20 minutes). Myocytes were washed with Calcium re-introduction solutions (100 µM, 400 µM and 900 µM: diluted in stopping buffer) with pelleting allowed between washes. Cells were mixed with plating medium containing: 10% FBS, Blebbistatin (25 µM), HEPEPS (10 mM), ATP (2 mM) and Primocin^TM^ (diluted 50 mg to 500 mL MEM, Invivogen, San Diego, CA. USA). Cells were placed on laminin coated coverslips, in 24 well plates and incubated at 37 °C for 2 hours. Plating medium was replaced by culture medium containing: 0.1% BSA, Blebbistatin (25 uM), HEPES (10 mM), ITS (250 uL of 100x stock) and Primocin^TM^.

### Picro-sirius red stain

Left ventricular sections (7 μm) were washed in Xylene followed by washes in 100% and 95% EtOH. Tissue was placed in Hematoxylin, followed by tap water wash and Picro-Sirius Red application. This was followed by washes with acidic water, 100% EtOH and Xylene. Finally, coverslips were mounted onto slides with Permount. Imaging was performed using an Olympus Provis Light Microscope at 10x magnification. Data is reported as collagen: tissue ratio.

### Fibroblast culture

Primary cardiac fibroblasts isolated from the male F-344 rat model were grown to 80–90% confluence and washed with HEPES BSS (100 μL/cm^2^), followed by Trypsin/EDTA (100 μL/cm^2^) for less than 5 minutes, and Trypsin Neutralization Solution (100 μL/cm^2^). Solution was then spun down for 3 minutes at 220 × g followed by resuspension in pre-warmed growth media. Cells were counted and stored in Cryo-SFM media (PromoCell, C-29910) at −80 °C for slow freezing for 24 hours and moved to −120 °C for long term storage. Frozen fibroblasts were thawed at 37 °C until no ice remained in the vial (~2 minutes). Fibroblasts were seeded between 5,000–10,000 cell/cm^2^. Media (PromoCell, C-23130) was replaced after 24 hours and every 2 days thereafter until 90% confluency was reached. Fibroblasts were treated with RLX (25 nM), TGFβ (2 ng/mL), Dkk (0.1 μg/mL), and Wnt1 (0.1 μg/mL).

### RT-PCR analysis

RNA was isolated (RNAEasy, Qiagen) and copied to cDNA (High Capacity Reverse Transcription kit, Applied Biosystems) according to manufacturer protocols. A Syber-green-based formulation (Absolute Sybr-Green, Thermo Fischer Scientific, Waltham, MA) was utilized for fluorescence-based kinetic real-time PCR using an Applied Biosystems model 7000 detection system (Applied Biosystems Inc., Foster City, CA). Expression levels of RNAs of interest were normalized to that of GAPDH using the ΔΔCt method, and reported relative to the mean of the WTV group. Primer pair sequences (forward and reverse for each target, listed 5′ to 3′) used for RT-PCR are as follows:

MMP-2: gcaccaccgaggattatgac, cacccacagtggacatagca;

MMP-9: cctctgcatgaagacgacataa, ggtcaggtttagagccacga;

αSMA: tggctgatggagtact-tc, gatagagaagccaggatg;

Wnt1: cctgcacctgcgactacag, ggttcatgaggaagcgtagg

GAPDH: agctggtcatcaatgggaa, atttgatgttagcgggatc.

### Western blot

Protein from aged rat lysates ± RLX (n = 3/group) was separated using pre-cast Mini-PROTEAN TGX 7.5% polyacrylamide gels and transferred to a PVDF membrane. Membranes were then probed for mouse anti-DKK1 (1:500, Santa Cruz sc374574) and rabbit anti-α-tubulin (1:1000, Abcam, ab4047). Membranes were developed using Pierce ECL Western Blotting Substrate (ThermoScientific, #32109) according to manufacturer instructions and developed by autoradiography. Quantification of the optical density of DKK1 or tubulin bands were performed using ImageJ software and DKK1 expression was normalized to α-tubulin.

### Statistical analysis

Comparisons between two groups were done using an unpaired, 2-tailed t-test, or the Mann-Whitney U test. Comparisons of three or more groups were done using ANOVA or Kruskal-Wallis tests. Nuclear β-catenin was compared using Chi-squared. All data is presented as mean ± SEM, unless otherwise noted. Statistical comparisons were performed using Graphpad Prism software. A value of *P* < 0.05 was considered to be statistically significant.

### Ethical approval and informed consent

1. An approval of experimental protocols is stated in the Methods section from the University of Pittsburgh. 2. All methods were carried out in accordance with NIH guidelines and the animal usage committee of the University of Pittsburgh. 3. There was no informed consent as this did not involve patients or human tissues.

## Supplementary information

Supplementary Material

## Data Availability

All data relevant to the manuscript are available for the reviewers to examine and should be manuscript be accepted for publication in *Scientific Reports* the authors assert that they submit these data in a supplemental data manuscript, or it will be added to the supplement.
