# Supplementary material for: Relaxin reverses maladaptive remodeling of the aged heart through Wnt-signaling
Source: Sci Rep. 2019 Dec 6;9:18545. doi: 10.1038/s41598-019-53867-y (PMC6897890; doi:10.1038/s41598-019-53867-y)
Supplement: Supplementary file 1 — Supplementary Material [file 41598_2019_53867_MOESM1_ESM.pdf]

Supplementary Material:

Relaxin reverses maladaptive remodeling of the aged heart through Wnt-signaling

Brian Martin[1,2], Beth Gabris[2], Amr F. Barakat MD[2], Brian Henry MD, Ph.D.[2] Marianna Giannini Ph.D. [†,2], Rajiv P. Reddy[2], Xuewen Wang MD, PhD[2#], Guillermo Romero Ph.D.[3] and Guy Salama Ph.D.[1, 2,*]Control experiments were carried out with young adult (9-months old) F-344 rats which were treated with Relaxin or with the vehicle as described in Methods.

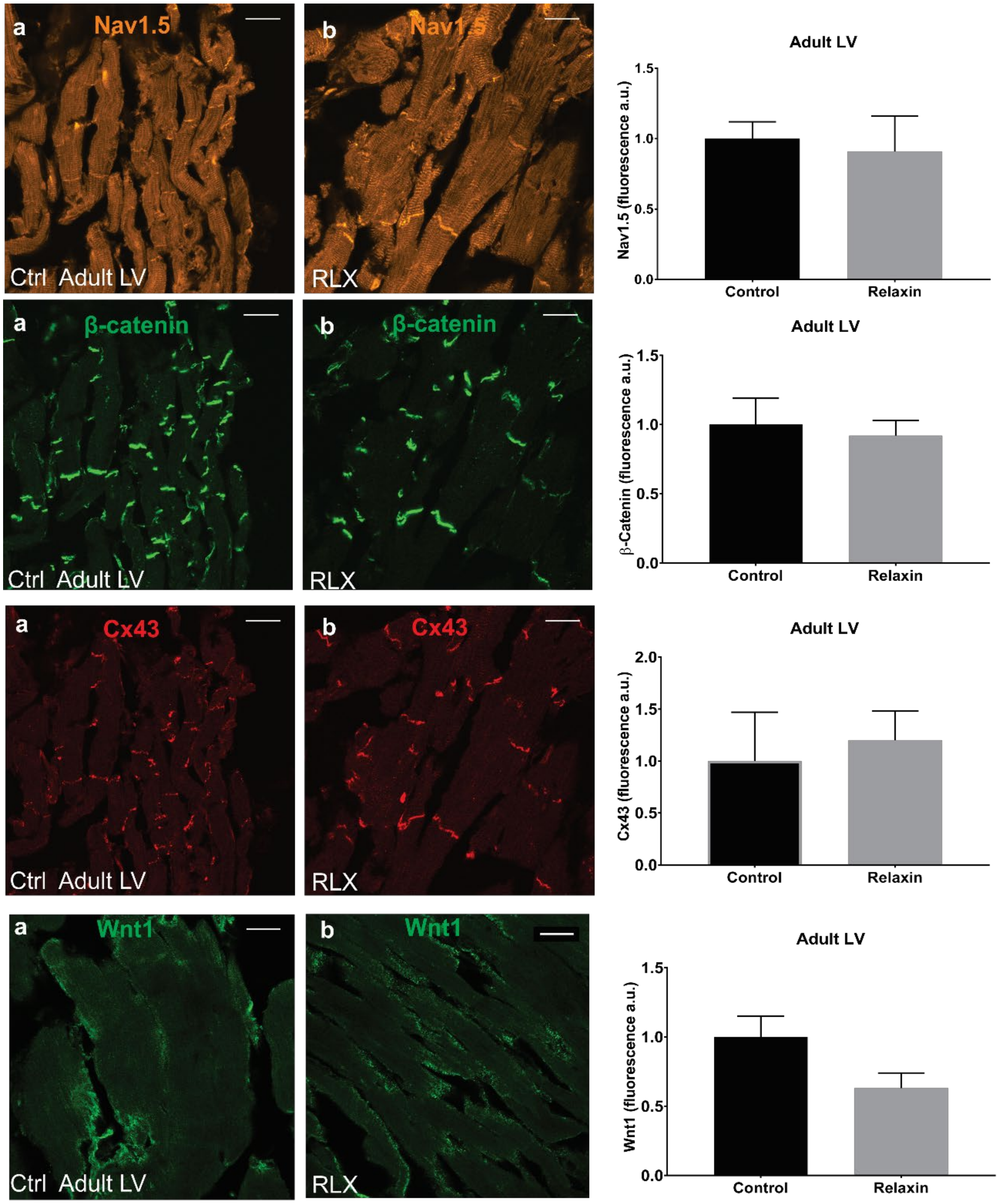

Supplementary Figure 1S

As shown in Figure 1S, Relaxin has no significant effects on the younger hearts which already express Nav1.5, β-catenin and connexin 43 abundantly and where β-catenin and Connexin 43 are primarily found at the intercalated disks and rarely on the lateral membranes. Similarly, young rat hearts express Wnt1 which is no significantly modified by Relaxin treatment. These results are consistent with our recent report on the genomic changes from RNA-seq analysis between young vs. old ventricles with or without the same Relaxin treatment, see Martin B, Gabris-Weber BA, Reddy R, Romero G, Chattopadhyay A, Salama G. Relaxin reverses inflammatory and immune
signals in aged hearts, PLOS ONE 13(1): e0190935.
https://doi.org/10.1371/journal.pone.0190935

Supplementary Figure 2S: Relaxin inhibits DKK1 expression in Aged LV: Full Blots. Relaxin significantly reduced DKK1 expression compared to control hearts. C = control. R = Relaxin. n = 3/group. Gels were probed for DKK1 expression (top), stripped and re-probed for, and normalized to, α-tubulin expression (bottom). Pure DKK1 protein was pipetted into lane 1 during DKK1 probing to ensure proper molecular weight of the DKK1 band from the aged animals.

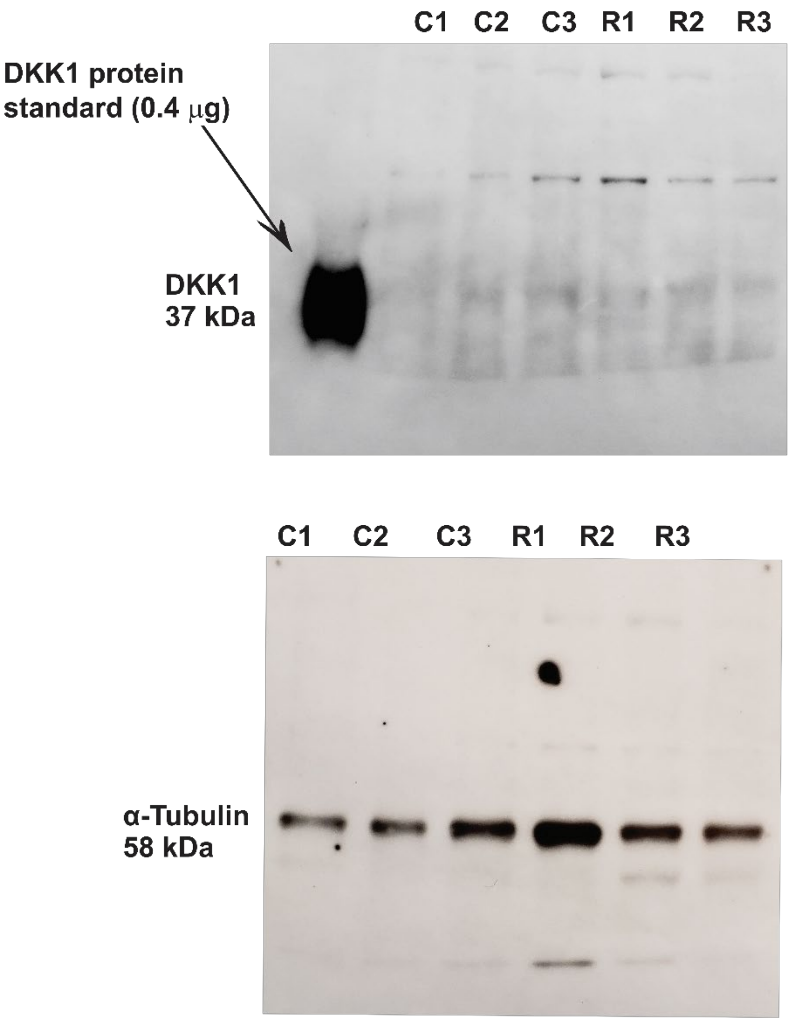

Supplementary Figure 2S
